# Label-Free miRNA-21 Analysis Based on Strand Displacement and Terminal Deoxynucleotidyl Transferase-Assisted Amplification Strategy

**DOI:** 10.3390/bios12050328

**Published:** 2022-05-12

**Authors:** Ying Yan, Han Zhao, Yukang Fang, Changbei Ma, Junxiang Chen

**Affiliations:** 1 School of Life Sciences, Central South University, Changsha 410013, China; yany2018@csu.edu.cn (Y.Y.); zhaohan202010@163.com (H.Z.); 8305200806@csu.edu.cn (Y.F.); macb2012@csu.edu.cn (C.M.); 2 Department of Stomatology, Shanghai Tenth People’s Hospital, School of Medicine, Tongji University, Shanghai 200072, China; 3 Department of Urology, The Second Xiangya Hospital of Central South University, Changsha 410011, China

**Keywords:** miRNA-21, dual amplification, terminal deoxynucleotidyl transferase, strand displacement amplification, thioflavin T

## Abstract

MicroRNAs (miRNAs) are regarded as a rising star in the biomedical industry. By monitoring slight increases in miRNA-21 levels, the possibilities of multi-type malignancy can be evaluated more precisely and earlier. However, the inconvenience and insensitivity of traditional methods for detecting miRNA-21 levels remains challenging. In this study, a partially complementary cDNA probe was designed to detect miRNA-21 with target-triggered dual amplification based on strand displacement amplification (SDA) and terminal deoxynucleotidyl transferase (TdT)-assisted amplification. In this system, the presence of miRNA-21 can hybridize with template DNA to initiate SDA, generating a large number of trigger molecules. With the assistance of TdT and dGTP, the released trigger DNA with 3′-OH terminal can be elongated to a superlong poly(guanine) sequence, and a notable fluorescence signal was observed in the presence of thioflavin T. By means of dual amplification strategy, the sensing platform showed a good response tomiRNA-21 with a detection limit of 1.7 pM (S/N = 3). Moreover, the specificity of this method was verified using a set of miRNA with sequence homologous to miRNA-21. In order to further explore its practical application capabilities, the expression of miRNA in different cell lines was quantitatively analyzed and compared with the qRT-PCR. The considerable results of this study suggest great potential for the application of the proposed approach in clinical diagnosis.

## 1. Introduction

Micro-RNA refers to a type of short non-coding endogenous RNAs with a length of approximately 22 nucleotides [1,2]. Ubiquitously distributed within mammalian bodies, micro-RNA has been shown to regulate a myriad of physiologic or pathologic processes. The growing achievements in understanding micro-RNA’s role in chronic diseases have make it well-known in the biomedical industry, especially for the improvement of anti-cancer measures [3]. Recently, with the popular concept of ‘liquid biopsy’, the importance and potential of micro-RNA detection in cancer management have been emphasized once again [4,5]. Large-scale population-based studies have also verified the value of micro-RNA in early screening for cancer [6,7,8]. However, challenges remain in translating micro-RNAs into application. For instance, due to the heterogeneity of the biologic roles of micro-RNA, detection of certain micro-RNAs may lead to contradictory results in indicating diseases [9,10]. Therefore, detection of micro-RNAs should focus on a target with less diverse functions. As a type of micro-RNA widely up-regulated in malignant diseases, miRNA-21 is an excellent target for detection [11]. By monitoring any slight increase of miRNA-21 levels, the possibilities of multi-type malignancy can be evaluated more precisely and earlier.

From the perspective of miRNA-21 analysis, various detective strategies and platforms have been developed [12]. Traditionally, real-time polymerase chain reaction (RT-PCR), northern blotting and microarrays have been widely utilized; however, these methods are usually universal assays for nucleic acids, and may increase false-positive rates when detecting micro-RNA in complex environments [13,14,15]. To solve this problem, highly specific probes based on the sequence of miRNA-21 for determination have been used. By modulating the complementary combination of probe and miRNA-21, a detective signal can be easily generated. This design strategy was named target-triggered detection and became a basic principle for micro-RNA analysis [16,17,18,19]. Another challenge is the inconvenience and insensitivity of traditional methods. Currently, processes such as liquid biopsy, detection at nanomole or even picomole level and large-scale sample screening are often required or involved [4]. Thus, the application of powerful amplification strategies will greatly enhance detection ability. In recent years, several representative strategies have been used in micro-RNA analysis, such as enzyme-mediated amplification, rolling circle amplification (RCA), strand displacement amplification (SDA), enzyme-assisted target recycling (EATR), terminal deoxynucleotidyl transferase (TdT)-assisted amplification, and non-enzyme amplification including catalyzed-hairpin assembly amplification (CHA) and hybridization chain reaction (HCR), [20,21,22,23,24,25,26]. Among these, SDA and TdT-assisted amplification have gained attention owing to their merits of high amplification efficiency. SDA uses a restriction enzyme to nick at a specific site and a polymerase to initiate a new cycle of replication from the 3′-OH end of the nick, which can exponentially amplify the target sequence [22]. TdT is a unique DNA polymerase that can catalyze the addition of dNTPs to the 3′-OH end of DNA molecules without template [20]. In a previous study, a highly sensitive determination of protein biomarker based on TdT-assisted amplification was achieved [27]. In this study, we aimed to improve the sensitivity of miRNA-21 detection by dual amplification.

Recently, the G-quadruplex/Thioflavin T (ThT) system has been widely used for signal generation in biochemical analysis. This system can exhibit remarkable fluorescence signal enhancement [23,28,29], and this characteristic makes ThT a label-free fluorescent indicator with broad applicative prospects for the construction of fluorescent biosensors. Compared to other fluorescence systems for biological analysis, the G-quadruplex-ThT fluorescence platform exhibits advantages of low cost, being label-free and having good biocompatibility.

In this study, to further strengthen the signal a multi-amplificative design was applied consisting of SDA and TdT-mediated amplification, with a G-quadruplex/ThT reporting system, for miRNA-21 analysis. To combine these three systems, several enzymes were used as tools. In our design, the presence of miRNA-21 can hybridize with template DNA to initiate SDA, generating a large number of trigger molecules. With the assistance of TdT and dGTP, the released trigger DNA with 3′-OH terminal was elongated to a superlong poly(guanine) sequence, so that a notable fluorescence signal was observed after ThT was added. By means of dual amplificative strategy and efficient reporting design, the sensing platform showed a good response towards miRNA-21 with a detection limit of 0.0017 nM (S/N = 3). In specificity analysis, a set of miRNAs with sequence homology to miRNA-21 was selected. In order to further explore its practical application capabilities, the expression of miRNA in different cell lines was quantitatively analyzed and compared with the qRT-PCR. All the results of this study suggest that the proposed approach has great potential in clinical diagnosis.

## 2. Materials and Methods

### 2.1. Materials and Reagents

Terminal deoxynucleotidyl transferase (TdT), deoxy-ribonucleoside triphosphates (dNTPs), deoxyadenosine triphosphate (dATP) and deoxyguanosine triphosphate (dGTP) were purchased from Takara Biotechnology Co., Ltd. (Dalian, China). Klenow fragment polymerase (3′-5′exo-, KF polymerase) and the nicking endonuclease Nt.BstNBI were bought from New England Biolabs Ltd. (Beverly, MA, USA). Tris [Tris-(hydroxy-methyl) aminomethane], hydrochloric acid (HCl), thioflavin T (ThT), sodium chloride (NaCl), magnesium chloride (MgCl_2_), were obtained from Sinopharm Chemical Reagent Co., Ltd. (Shanghai, China). DNA and RNA sequences were synthesized by Sangon Biotechnology Co., Ltd. (Shanghai, China). All other reagents were of analytical reagent grades. Ultrapure water (18.2 MΩ.cm) used in the experiments was obtained from a Milli-Q water purification system (Millipore Corp, Bedford, MA, USA). The DNA sequences and RNA sequence list were as follows: miRNA-21, 5′-UAG CUU AUC AGA CUG AUG UUG A-3′; cDNA: ATA TCA GCG ATC ACC CAT GTT ACT CTC TAA CAG ACT CTC AAC ATC AGT CTG ATA AGC TA-3′. The reaction buffer used in this study contained two components: (1) Tris-HCl buffer (50 mM Tris-HCl, 100 mM NaCl, 10 mM MgCl_2_, pH 7.9), (2) 1 × TdT buffer.

### 2.2. Apparatus

The fluorescence measurements were performed on a Hitachi F-2700 fluorescence spectrophotometer (Hitachi Ltd., Hitachi, Japan). The excitation wavelength was set at 425 nm and the fluorescence emission spectra were collected at wavelengths ranging from 450 to 550 nm at room temperature. All experiments were repeated at least three times. The slit widths of emission and excitation were both set at 5 nm.

### 2.3. Optimization of the Experimental Conditions

All experimental conditions were optimized, including the concentrations of cDNA, KF polymerase, Nt.BstNBI, TdT, and the reaction time of TdT. The concentration range of cDNA was 20–80 nM. The selected KF polymerase concentration was 50–150 U/mL. The Nt.BstNBI concentration was 20–60 U/mL. The selected concentration of TdT was 70–350 U/mL. The TdT reaction time was 120–240 min. When optimizing the experimental conditions, only one condition was changed at a time, and the other conditions remained unchanged.

### 2.4. MiRNA-21 Assay

The determination of miRNA-21 was conducted as described below. Initially, 40 nM cDNA and different concentrations of miRNA-21 ranging from 0 to 200 nM were added to a mixed buffered solution containing 20 μL buffer A (50 mM Tris-HCl, 100 mM NaCl, 10 mM MgCl_2_, pH 7.9). The mixed buffer A was incubated at 37 °C for 40 min to form partially complementary duplex. Then, 100 U/mL KF polymerase, 40 U/mL Nt.BstNBI nicking endonuclease, and 0.1 mM dNTPs were added to the resulting solution A at 37 °C for 2 h to generate trigger DNA. Then, the digestion and the extension reaction were terminated by incubating at 80 °C for 20 min. Later, 210 U/mL TdT, 0.4 mM dATP, 0.6 mM dGTP and 20 μL 1 × TdT buffer were added and incubated in the reaction system for 180 min at 37 °C. Finally, 60 μL buffer A (50 mM Tris-HCl, 100 mM NaCl, 10 mM MgCl_2_, pH 7.9) and 8 mM ThT were mixed with the solution to give a final volume of 100 μL, and vigorously stirred to react at 25 °C for 10 min. The fluorescence emission spectrum was recorded at room temperature using an F-2700 fluorescence spectrophotometer.

### 2.5. Selectivity Assay

In order to explore the selectivity of the proposed system, several interfering miRNAs including miRNA122, miRNA141, and miRNA155 were chosen as comparisons. The protocol was similar to that used for the concentration measurements of miRNA-21. N.C stand for random single-stranded oligonucleotide sequences. Blank samples without miRNA-21 were also prepared and used as standards.

### 2.6. Assay for MiRNA-21 in Biologic Sample

Total RNA of HEK293, HeLa, and MCF-7 cells were used as real samples. Total RNA was extracted using a Tissue RNA Purification Kit PLUS (EZBioscience, Roseville, MN, USA) from the above-mentioned different types of cells. The procedures of kit operation, sample homogenization, RNA binding (through column), column washing, and RNA elution to extract RNA were strictly followed. Then, the RNA extracted from the above cells was dissolved in DEPC water, and the extraction solution was used to detect miRNA-21 by the newly developed detection method constructed in this article and by the traditional qRT-PCR method. The protocol for performing qRT-PCR was: denaturation (5 min, 95 °C), and 40 amplification cycles (10 s at 95 °C, and 30 s at 60 °C). Each assay was performed in triplicate.

## 3. Results

### 3.1. Principle of the MiRNA-21 Detection

**Scheme 1:**
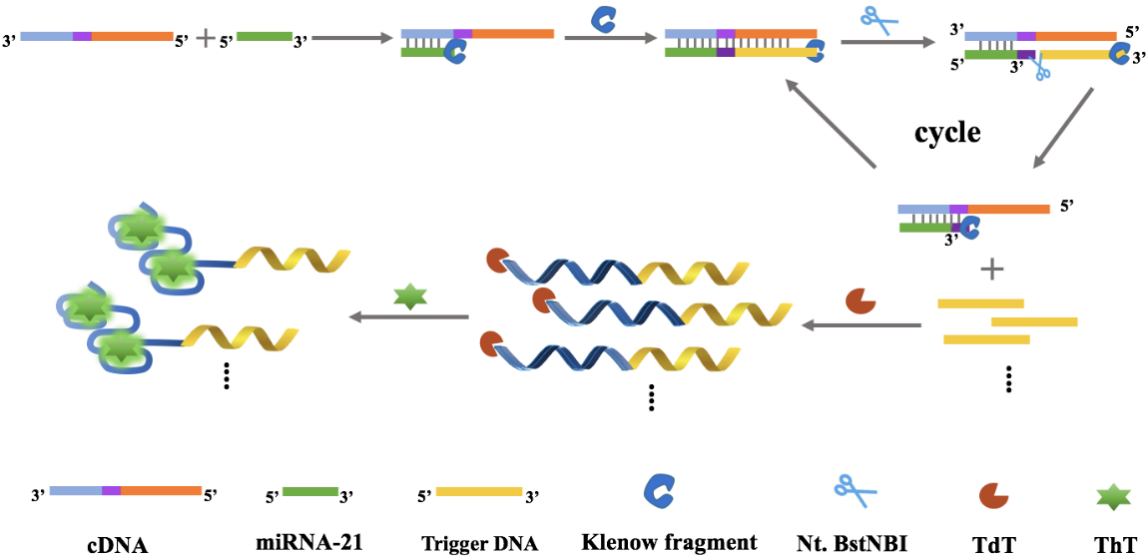
Schematic of miRNA-21 detection based on isothermal strand displacement amplification strategy and G-quadruplexe-thioflavin T system.

In this study, a dual amplificative system for miRNA assay was developed by combining SDAs and TdT-mediated signal-on fluorescence reporting. As depicted in Scheme 1, a DNA probe (named cDNA) was designed to recognize miRNA-21 and trigger the following reactions, comprising three parts: the miRNA hybridization region, the recognition region for the nicking endonuclease Nt.BstNBI, and the DNA strand amplification region. In the presence of miRNA-21, it was able to hybridize with the target to form partially complementary DNA/RNA duplex. After the addition of KF polymerase and dNTPs, miRNA was extended from its 5′-terminal and generated a double-stranded structure with the recognition site for Nt.BstNBI (5′-GAGTC-3′). Nt.BstNBI then produced a single-strand gap at four bases downstream of the recognition site, yielding a new replication site, and trigger DNA was released and the target recycled. Simultaneously, a break at the 3′-end of nucleic acid strand could be extended again. Through successive polymerization, cleavage and strand replacement reactions, sufficient trigger DNA was produced and was involved in the following reaction. After that, the released trigger DNA acted as primer to participate in the TdT-mediated elongation reaction. TdT could catalyze the addition of poly(G) and poly(A) tails to the 3′-OH end of single-stranded DNA (trigger DNA), forming G/A rich sequences. They can fold into the G-quadruplex structure and combine with ThT to form G-quadruplex/ThT complex, resulting in a remarkable fluorescence signal which can be observed. In the absence of miRNA, cDNA with 3′-P terminal cannot be extended by TdT, and G/A-rich sequence is not generated, accompanied by the quenching fluorescence signal. Therefore, depending on the change in the fluorescence signal, a “turn-on” detection strategy of miRNA was obtained.

The design principle of this work can extensively improve the signal based on the following facts: (1) A recycling process was involved in SDA. The recycling part of target is not merely miRNA-21, the analyte, which is the single-strand RNA and may be easily degraded under improper conditions. Instead, upon the reaction, the cDNA captures miRNA-21 and its hybrids, making sure that the analyte is unlikely to be degraded during the following reactions and trigger the following amplification. (2) A dual amplificative design consisting of SDA and TdT-mediated elongation reaction was applied. The enzymes involved in this system play key roles in amplification, and their high specificity ensures that the amplification to be worked is in order and free of interference. (3) The G-quadruplex/ThT complex functions as the signal reporting system, and it has superior signal intensity compared to other label-free methods, further strengthening the signal indicating ability of this system.

### 3.2. Feasibility of MiRNA-21 Assay

**Figure 1:**
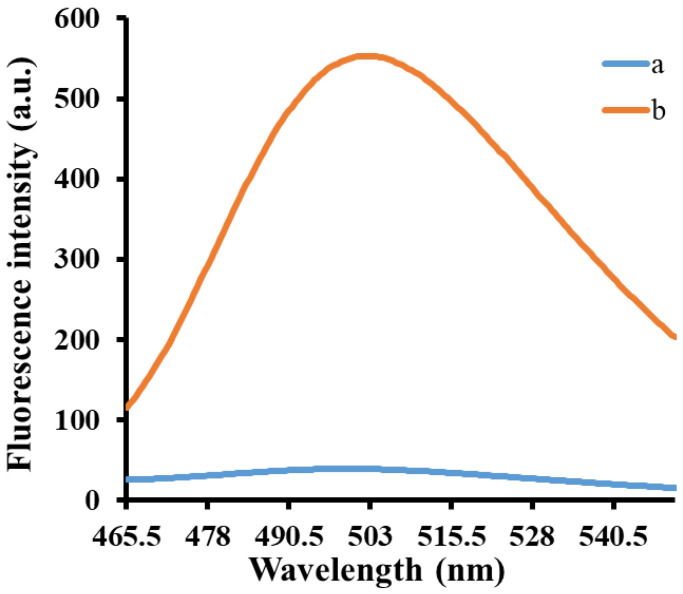
The feasibility test of the proposed strategy. (**a**) cDNA + KF polymerase + NtBstNBI + TdT; (**b**) cDNA + miRNA-21 + KF polymerase + NtBstNBI + TdT. Concentrations of cDNA, KF polymerase, NtBstNBI and TdT were 60 nM, 100 U/mL, 40 U/mL and 280 U/mL, respectively.

The feasibility of the proposed method was verified by measuring the fluorescence emission spectra of the miRNA-21 detection system under different conditions. As shown in Figure 1, the isothermal amplification was not initiated in the absence of miRNA-21, and the addition of TdT and dGTP did not produce a poly(guanine) sequence. Therefore, only a very low fluorescence signal was observed (curve a). In contrast, miRNA-21 could hybridize with the template cDNA and initiate isothermal strand displacement amplification in the presence of miRNA-21. With the assistance of TdT and dGTP, the released trigger DNA with 3′-OH terminal resulted in the super-long poly(guanine) sequence, leading to the obvious enhancement in the fluorescence signal after adding ThT (curve b). Therefore, these results show that the proposed detection system has good feasibility and can be used for subsequent experiments.

### 3.3. Optimization of Experimental Conditions

**Figure 2:**
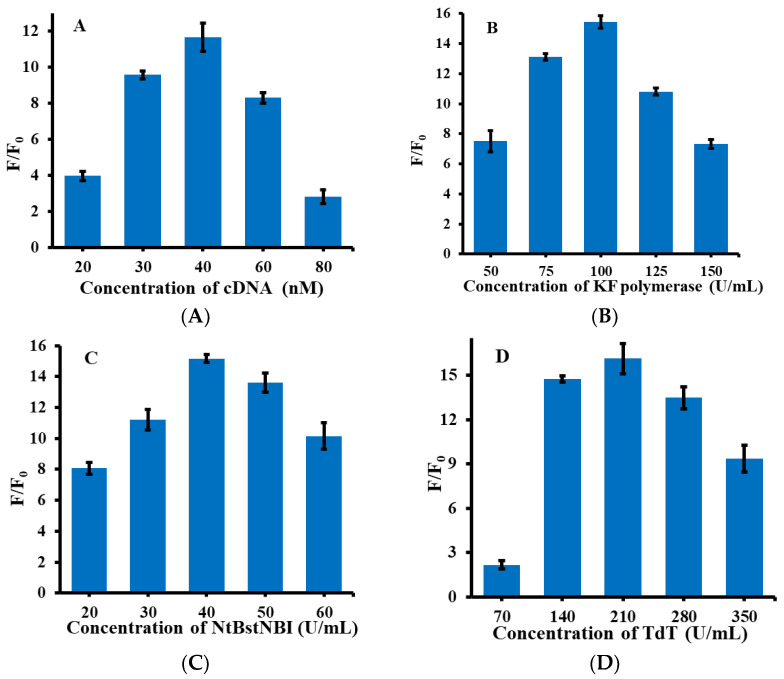

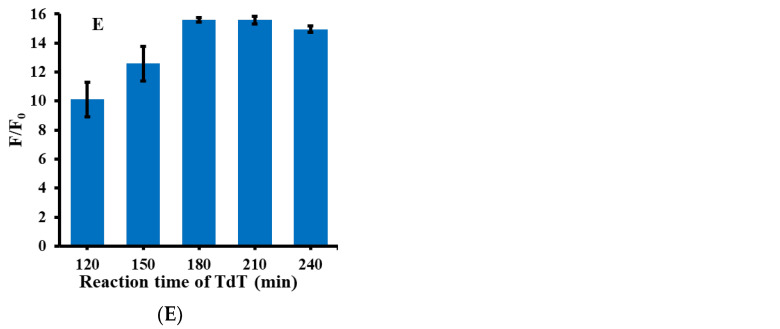
Optimization of the experimental conditions for the detection system of miRNA-21. (**A**) Concentrations of cDNA (20, 30, 40, 60, 80 nM); (**B**) Concentration of KF polymerase (50, 75, 100, 125, 150 U/mL); (**C**) Concentration of NtBstNBI (20, 30,40, 50, 60 U/mL); (**D**) Concentration of TdT (70, 140, 210, 280, 350 U/mL); (**E**) TdT reaction time (120, 150, 180, 210, 240 min). The concentration of the other reagents remained the same when each experimental condition was optimized.

As shown in Figure 2, in order to obtain a highly sensitive detection system for miRNA-21, several important conditions were optimized during the reaction process, including the concentration of cDNA, KF polymerase, Nt.BstNBI, TdT, and the reaction time of TdT. F/F0 in the figure refers to the ratio of fluorescence intensity in the presence and absence of miRNA-21. Firstly, cDNA was optimized with a concentration in the range of 20–80 nM. As shown in Figure 2A, when the cDNA concentration was 40 nM, the F/F0 ratio was the highest. Therefore, 40 nM was selected as the optimum reaction condition for cDNA. Secondly, the concentration of KF polymerase was optimized, as shown in Figure 2B. Within the concentration range of 50–150 U/mL, 100 U/mL was the optimal reaction concentration of KF polymerase. Thirdly, the concentration of Nt.BstNBI was optimized, and Nt.BstNBI with a concentration range of 20–60 U/mL was selected for the optimization experiments. As shown in Figure 2C, the reaction effect was best when the Nt.BstNBI concentration was 40 U/mL, so 40 U/mL Nt.BstNBI was selected as the optimum experimental condition. Then, the reaction concentration of TdT was further optimized, and the selected concentration of TdT was 70–350 U/mL. The results can be seen from Figure 2D; the ratio of fluorescence intensity was highest when TdT was 210 U/mL, so 210 U/mL was selected as the optimal condition for TdT. Finally, the optimal incubation time of TdT was investigated. The TdT reaction time was in the range 120–240 min. Because TdT directly affects the formation of poly(guanine) sequence, the reaction time of TdT is also an important factor in the detection system. As shown in Figure 2F, when the reaction time reached 180 min, the value of F/F0 remained stable and almost never increased. Therefore, 180 min was selected as the optimum condition for the subsequent experiments.

### 3.4. Quantitative Measurement of MiRNA-21

**Figure 3:**
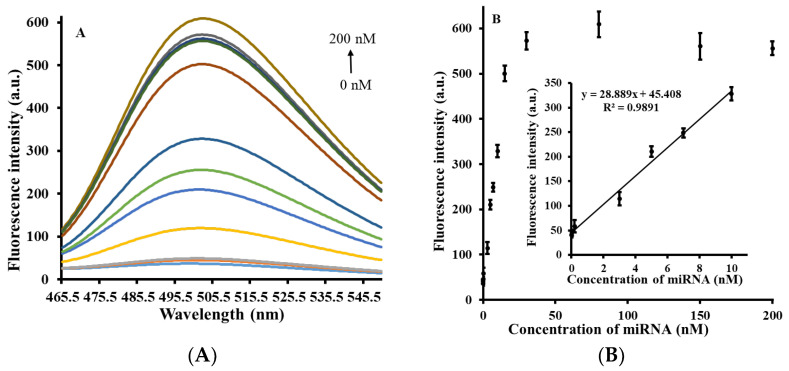
(**A**) Fluorescence emission spectra of the biosensor with different concentrations of miRNA-21 (0, 0.002, 0.02, 3, 5, 7, 10, 15, 30, 80, 150, 200 nM) added. (**B**) Graph of correlation between miRNA-21 concentration and fluorescence intensity. The inset shows that miRNA-21 concentration is linearly correlated with fluorescence intensity at 0.002–10 nM. The error reflects three separate measurements.

Under the above optimal experimental conditions, the miRNA-21 concentrations in the range of 0–200 nM (0, 0.002, 0.02, 3, 5, 7, 10, 15, 30, 80, 150, 200 nM) were selected for quantitative analysis. Figure 3A shows the fluorescence emission spectra of miRNA-21 at varying concentrations, with different colored curves representing different concentrations, indicating that with increasing miRNA-21 concentration, fluorescence intensity gradually increases. This is because the addition of miRNA-21 promotes the chain substitution reaction and formation of the poly(guanine) sequence, which leads to an increase in fluorescence intensity. Figure 3B shows the correlation graph of fluorescence intensity and miRNA-21 concentration obtained on the basis of Figure 3A. The inset of Figure 3B shows that miRNA-21 concentration in the range of 0.002–10 nM has a linear correlation with fluorescence intensity (R^2^ = 0.9891). The linear correlation equation is Y = 28.889X + 45.408, where Y represents the fluorescence intensity and X represents the concentration of miRNA-21. Finally, based on the calculation of the 3σ rule, the detection limit (LOD) of this strategy was obtained as 1.7 pM, which can compete with the majority of miRNA detection methods reported in recent years (Table 1).

### 3.5. Selectivity of the MiRNA-21 Assay

**Figure 4:**
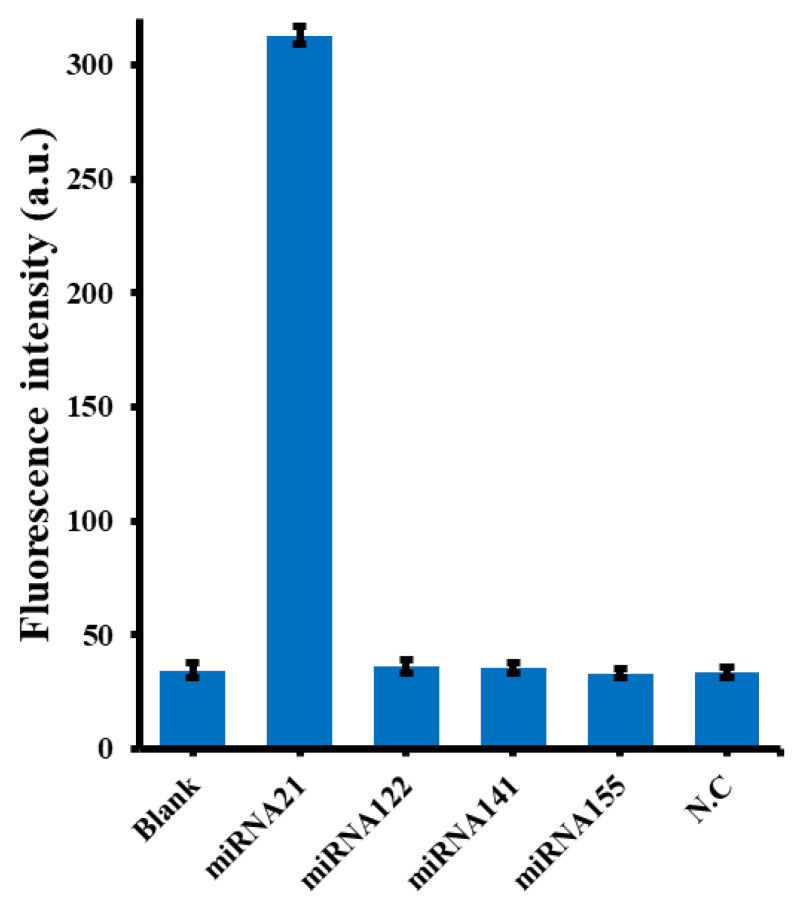
Specificity of the proposed sensor for miRNA-21 compared to other interfering RNA strands. The concentration of miRNA-21 was 10 nM, the concentrations of each interfering RNA strand (miRNA122, miRNA141, miRNA155 or N.C) were 10 nM.

As is mentioned above, practical sampling for miRNA-21 analysis can often be subject to interference from other analogues, especially other micro-RNA [10]. As shown in Figure 4, fluorescence intensity was highest only when miRNA-21 was added, while fluorescence intensity was almost the same as that of the blank control group in the presence of other interfering RNAs. This result also confirmed the selectivity of miRNA-21 in the dual amplificative reaction of the system, as expected, and also confirmed the good specificity of our proposed method for the detection of miRNA-21.

### 3.6. Application of the Method in the Determination of Biological Systems

The presence of miRNA is almost ubiquitous within the human body [2]. Methods of miRNA analysis should be examined both in extracellular and intracellular samples [4]. To achieve this, the application prospect of the proposed method was first validated in extracellular samples by performing recovery experiments, in which three miRNAs of different concentrations (3 nM, 5 nM and 7 nM) were recovered from 5% diluted serum using our newly established method. The experimental results are listed in Table 2. The recoveries of different concentrations of miRNA-21 in the diluted serum were 94.45% at 3 nM, 105.8% at 5 nM, and 98.26% at 7 nM. The RSDs were 9.6% at 3 nM, 9.55% at 5 nM, and 1.49% at 7 nM. These results indicate that our method showed good application in serum dilution.

**Figure 5:**
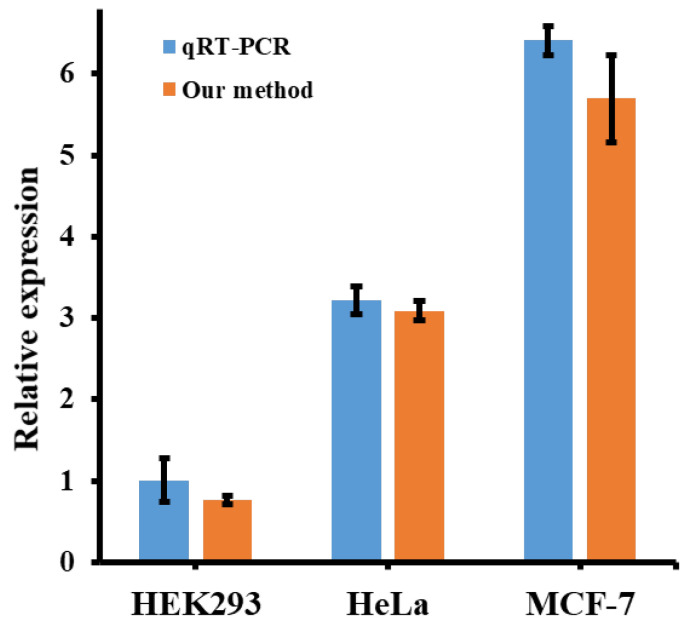
Histogram of miRNA-21 expression levels detected in MCF-7, HeLa, and HEK293 cells. Concentrations of cDNA, KF polymerase, NtBstNBI and TdT were 40 nM, 100 U/mL, 40 U/mL and 210 U/mL, respectively.

In order to make our conclusions more convincing, miRNA-21 was further tested in different cells’ lyses. Three types of cells (HEK293, HeLa, and MCF-7) were selected to detect miRNA-21 expression levels. According to the literature, the expression of miRNA-21 in these cells MCF-7, HeLa and HeK293 is in descending order, which is representative for intracellular samples [23,38]. The experimental results, shown in the orange bar in Figure 5, were further confirmed by a gradual increase in the expression of miRNA-21 in HEK293, HeLa, and MCF-7 cells. In addition, to verify the sensitivity of miRNA-21 detection, the proposed method was compared with the qRT-PCR results. As shown in the blue bar chart in Figure 5, the relative expression of miRNA-21 detected by our method showed a similar trend to the results detected by qRT-PCR kit, under a certain error allowance. The above two experiments proved that the proposed miRNA-21 detection strategy shows very similar performance compared with the traditional methods, and exhibits good practical application prospects.

## 4. Conclusions

To sum up, a highly sensitive and selective sensor platform was developed for the detection of miRNA-21, based on isothermal strand displacement amplification strategy and the G-quadruplexe-thioflavin T system. Calculation based on the 3σ rule resulted in a low detection limit of 1.7 pM with a linear range of 0.002–10 nM. Finally, and importantly, this newly developed method for the detection of miRNA-21 was well validated in the selective assay, serum recovery assay, and actual cell sample assay. Therefore, we can predict that the method proposed in this study is able to make a definite contribution to the detection of miRNA-21, and play a potential part in early clinical diagnosis and targeted therapy of malignant tumors in the future.

## Figures and Tables

**Table 1:** Comparison of different methods for the detection of miRNA-21.

Methods	Materials	LOD (nM)	Dynamic Range (pM)	Reference
Colorimetric	Ag/Pt nanocluster	0.01–1	4.1	[30]
Electrochemical	Au nanoparticle	0.001–1	0.3	[31]
Electrochemical	Au nanoparticle	0.002–10	6.8	[32]
Electrochemical	MnO_2_ nanoflake	0.4–100	250.0	[33]
Fluorescence	Ag nanocluster	0.1–8000	60.0	[34]
Fluorescence	2-Aminopurine/ThT	0.5–50	72.0	[21]
Fluorescence	DNA nanomachine	0.1–10	80.0	[35]
Fluorescence	Framework nucleic acid	0–500	40	[36]
Fluorescence	DNA nanowire	0.01–1	1.2	[37]
Fluorescence	ThT	0.002–10	1.7	This work

**Table 2:** Experimental recovery results of human serum samples (*n* = 3).

Sample	Added (nM)	Found (nM)	Recovery (%)	RSD (%)
1	3	2.834 ± 0.308	94.45	9.6
2	5	5.290 ± 0.571	105.8	9.55
3	7	6.878 ± 0.103	98.26	1.49

## Data Availability

Not applicable.
